# Association Between Pre-hospital Medication Use and Outcomes in Patients Hospitalized for COVID-19 Pneumonia: A Multicenter Observational Study in the Netherlands

**DOI:** 10.7759/cureus.99243

**Published:** 2025-12-15

**Authors:** Letao Li, Joyce Goris-de Geus, Iris de Leeuw, Floortje Kanits, Jaccoline Zegers, Arjan van Zadelhoff, Noud Buenen, Thijs Clemens David Rettig, Monique P. L Hoir, Geerke Duijzer, Johanna Marianne Geleijnse, Henrik Endeman

**Affiliations:** 1 Pharmacy, Erasmus University Medical Center, Rotterdam, NLD; 2 Pediatrics, ZorgSaam Hospital, Terneuzen, NLD; 3 Public Health Service, Municipal Health Service of North- and East-Gelderland (GGD NOG), Warnsveld, NLD; 4 Consumption and Healthy Lifestyles, Wageningen University and Research, Wageningen, NLD; 5 Human Nutrition and Health, Wageningen University and Research, Wageningen, NLD; 6 Intensive Care, Erasmus University Medical Center, Rotterdam, NLD; 7 Emergency Medicine, Maxima Medical Centre, Veldhoven, NLD; 8 Emergency Medicine, Bernhoven Hospital, Uden, NLD; 9 Intensive Care, Anesthesiology and Pain Medicine, Amphia Hospital, Breda, NLD; 10 Intensive Care, Our Lady Hospital, Amsterdam, NLD

**Keywords:** covid-19, icu, mortality, polypharmacy, pre-infection medication

## Abstract

Background

Home medication use may influence the clinical trajectory of COVID‑19, yet its association with adverse outcomes such as intensive care unit (ICU) admission and in‑hospital death remains unclear. We aim to explore whether pre-admission use of commonly prescribed medication - anticoagulants, antiplatelet agents, renin-angiotensin-aldosterone system (RAAS) inhibitors, corticosteroids, nonsteroidal anti-inflammatory drugs (NSAIDs), immunosuppressants, hydroxychloroquine, and statins - is associated with ICU admission and in-hospital mortality among adults hospitalized with COVID-19 across three Dutch hospitals.

Methods

We conducted a retrospective analysis using data from three Dutch hospitals containing variables including 22 medications. After recoding variables as factors, we performed descriptive analyses to assess basic associations. We then fitted multivariate logistic regression models correcting for patients' characteristics (age, gender) and disease status (diabetes, hypertension) to identify which medications were significantly associated with ICU admission and death.

Results

Composite variables representing anticoagulants, aspirin, and corticosteroids were independently associated with reduced odds of ICU admission, whereas hydroxychloroquine use was linked to increased risk. In contrast, for in-hospital mortality as an outcome, the use of heparin‑related medications was associated with increased odds of death.

Conclusion

Our observational analysis indicates that pre‑hospitalization medication patterns are significantly associated with COVID‑19 outcomes. While some medication groupings appear protective with respect to ICU admission, others are linked with increased mortality. These findings underscore the importance of further research to disentangle correlation from causation and to guide therapeutic decision‑making in COVID‑19 management.

## Introduction

Coronavirus disease 2019 (COVID-19), caused by SARS-CoV-2, continues to impose a substantial global burden. As of April 2025, the World Health Organization (WHO) has recorded 778 million confirmed infections and 7 million deaths worldwide [1]. Among hospitalized patients, clinical outcomes vary widely - from recovery to respiratory failure, multi-organ dysfunction, and death. The risk factors, such as advanced age, male sex, obesity, and chronic comorbidities (e.g., diabetes, cardiovascular disease, chronic kidney disease, and chronic obstructive pulmonary disease), increase the likelihood of complications [2-5].

Because these comorbidities often require long-term treatment, most patients are already taking one or more chronic medications when they are admitted to the hospital. There has been intense interest in whether common home medications might modulate the severity of COVID-19 [6]. Since early 2020, many investigators have explored whether pre-admission use of renin-angiotensin system blockers [7], statins [8,9], antiplatelet agents [10-13], nonsteroidal anti-inflammatory drugs (NSAIDs) [14,15], anticoagulants [16-18], glucocorticoids [19], or other classes of medications [20-23] alters COVID-19 severity. However, findings remain inconsistent - partly because most studies have focused on a single drug class and did not adjust for concomitant medications and comorbidities.

COVID patients, especially the elderly, rarely take medications alone; polypharmacy is common. However, little is known about the combined impact of multiple chronic medications on outcomes in hospitalized COVID-19 patients. This is important in clinical practice when a patient is admitted to the hospital; clinicians must optimize the treatment plan, change or continue the current treatment, so that the patient can get the greatest benefit. In addition, while current studies have focused mainly on drugs initiated after COVID-19 diagnosis, the prognostic value of drugs used before COVID-19 infection has received relatively limited attention.

Given the inadequacy of medication research for patients before COVID-19 infection and the potential confounding introduced by concomitant medications, we conducted a retrospective observational study of patients hospitalized with COVID-19 across three centers in the Netherlands. The aim is to evaluate the association between commonly prescribed pre-infection medications (anticoagulants, antiplatelet, hydroxychloquine, renin-angiotensin-aldosterone system (RAAS) inhibitors, NSAIDs, steroids, immunosuppressants, and statins) and the risk of critical outcomes after hospitalization (ICU admission and in-hospital mortality). Understanding these relationships could inform clinical management by identifying patients who might be at higher risk and informing decisions about continuing or temporarily discontinuing specific medications after hospitalization.

## Materials and methods

Study design and data source

This retrospective study included patients with confirmed COVID-19 who were admitted between February 20 and June 24, 2020, to three Dutch hospitals: Amphia Hospital (Breda), Erasmus University Medical Center (Rotterdam), and Bernhoven Hospital (Uden). Patients were excluded based on the following criteria: COVID-19 testing not performed or a negative result, not admitted to a hospital ward, admission for an unrelated medical procedure, an undocumented medication history, missing age data, or an inter-hospital transfer during the admission period. We collected data on pre-admission medication use, hospital admission dates, demographics, and health status from the electronic medical records of the included patients. Information on the clinical indications for pre-admission medications (e.g., atrial fibrillation for anticoagulants, autoimmune disease for immunosuppressants) was not available in the electronic records we accessed; therefore, confounding by indication cannot be excluded. Since all data were fully anonymized, the requirement for informed consent was waived. The study protocol was reviewed by the Medical Ethics Committee of Erasmus MC University Medical Center, which determined that the study does not fall under the Dutch Medical Research Involving Human Subjects Act (WMO).

Pre-admission medications

This study focused on commonly used pre-admission medications collected from the electronic health record (EHR), which were broadly classified as follows: (1) Anticoagulants, including heparin-like agents and oral anticoagulants. Oral anticoagulants were further subdivided into direct oral anticoagulants (DOACs) - such as the direct thrombin inhibitor dabigatran and direct factor Xa inhibitors like apixaban - and vitamin K antagonists (VKAs); (2) Platelet aggregation inhibitors (PAIs), such as clopidogrel and ticagrelor, and acetylsalicylic acid (aspirin); (3) RAAS inhibitors, including angiotensin-converting enzyme (ACE) inhibitors and angiotensin-II receptor blockers (ARBs); (4) NSAIDs, including non-selective COX-1 and COX-2 inhibitors (e.g., acetates, propionates); (5) Glucocorticoids, including systemic and inhaled glucocorticoids, (6) Immunosuppressive medications, including selective immunosuppressants, calcineurin inhibitors, and others; (7) Disease-modifying antirheumatic drug (DMARD) including hydroxychloroquine (HCQ); and (8) Other medications, such as lipid-lowering agents. Medication exposure was recorded as “use at admission (yes/no)” based on the medication checklist at hospital entry. The dose, formulation, duration of prior use, and adherence were not available for analysis.

Statistical analysis

Statistical analysis began with descriptive statistics for the study cohort. Continuous variables, such as age, were categorized for specific analyses (e.g., age ≥65 vs. <65 years). Categorical variables, including sex, comorbidities, medication use, and outcomes, were presented as counts and percentages. We then performed bivariate analyses to compare characteristics between key outcome groups (ICU admission vs. no ICU admission, and in-hospital mortality vs. survival). For these comparisons, we utilized Chi-square tests or Fisher’s exact tests (where cell counts were small) for categorical variables. These unadjusted analyses identified potential associations between pre-admission medications and outcomes. To assess independent associations while controlling for potential confounders (age, sex, diabetes, and hypertension), we constructed multivariable logistic regression models for each outcome: ICU admission and in-hospital mortality. Each initial model included these key characteristics along with all pre-admission medication variables of interest. A backward stepwise elimination procedure was applied to derive a parsimonious final model for each outcome. The results from these logistic regression models are presented as odds ratios (ORs) with their corresponding 95% confidence intervals (CIs) and p-values. Multivariable logistic regression analyses were performed using the glm() function (family = binomial) in R version 4.3.2 (R Foundation for Statistical Computing, Vienna, Austria). The multicollinearity of the factors was tested using vif() function.

## Results

A total of 1,153 patients were assessed for eligibility, specifically, among patients assessed for eligibility, we excluded those not COVID-19 tested/negative (n=69), not admitted to a ward (n=99), admitted for non-COVID procedures (n=14), with undocumented medication history (n=30), unknown age (n=2), or transferred between study hospitals (n=25). After applying the exclusion criteria, 914 COVID-19 patients were included for analysis. A total of 597 (65%) were male, and 615 (67%) were aged 65 years and older. Regarding comorbidities, 393 patients (43%) had hypertension, 364 (40%) had cardiovascular disease, and 233 (25%) had diabetes. In terms of outcomes, 288 patients (32%) required ICU admission during their hospitalization, and 274 (30%) died in the hospital. Detailed baseline characteristics of the study cohort are presented in Table 1.

**Table 1:** Patient characteristics at admission Continuous variables are presented as median (range) and categorical variables as n (%).

Characteristics	N=914
Age (years, range)	71 (16-96)
Body mass index (BMI) (kg/m^2^)	27.5 (16-54)
Comorbidities, n (%)	
Diabetes	233 (26)
Pulmonary disease	264 (28.9)
Cardiovascular diseases	364 (39.8)
Hypertension	393 (43)
Thromboembolism	42 (5)
COVID-19 outcome, n (%)	
ICU admission	288 (31.5)
In-hospital mortality	274 (30.0)

Regarding pre-admission medication use, 129 patients (14%) were receiving anticoagulants; 219 patients (24%) used antiplatelet therapy. Furthermore, 271 patients (30%) used ACE inhibitors or ARBs; 39 (4.3%) used at least one type of NSAID; 118 patients (13%) used systemic or inhaled corticosteroids; and 29 patients (3%) used immunosuppressants. Additionally, nine patients (1.0%) were taking HCQ. Statins represented the most frequently used medication group, with 345 patients (38%) receiving it. Detailed summaries of medication use are presented in Table 2.

**Table 2:** Medications used prior to COVID-19 infection NSAIDs: nonsteroidal anti-inflammatory drugs; ACE: angiotensin-converting enzyme

Drug classes	Medication	Patients, n (%)
Anticoagulants	Heparin-like agents	6 (1)
	Vitamin K antagonists	79 (9)
	Direct factor Xa inhibitors	34 (4)
	Direct thrombin inhibitors	11 (1)
Antiplatelet	Platelet aggregation inhibitors	97 (11)
	Aspirin	139 (15)
Disease-modifying antirheumatic drug (DMARD)	Hydroxychloroquine	9 (1)
Renin-angiotensin-aldosterone system (RAAS) inhibitors	ACE inhibitors	147 (16)
	Angiotensin II antagonists (ARBs)	126 (14)
NSAIDs	NSAIDs, pyrazoline derivatives	0 (0)
	NSAIDs, acetic acid derivatives, and related compounds	12 (1)
	NSAIDs, oxicam derivatives	6 (1)
	NSAIDs, propionic acid derivatives	17 (2)
	NSAIDs, coxibs	6 (1)
Steroids	Corticosteroids, systemic glucocorticoids	76 (8)
	Corticosteroids, inhaled	55 (3)
Immunosuppressants	Immunosuppressants, selective	7 (1)
	Immunosuppressants, calcineurin inhibitors	8 (1)
	Immunosuppressants, other	15 (2)
Lipid-lowering agents	Statins	345 (38)

The univariate associations between baseline characteristics, pre-admission medications, and ICU admission are presented in Table 3. Among medications, the use of VKAs and systemic corticosteroids was associated with a lower likelihood of ICU admission. In contrast, HCQ use was associated with an increased likelihood.

**Table 3:** Univariate analysis of prior medication and ICU admission DOACs: direct oral anticoagulants; ACE: angiotensin-converting enzyme; ARBs: angiotensin-II receptor blockers; OR: odds ratio; CI: confidence interval

Predictor	Test	OR with CI (95%)	P-value
Heparin-like substances	Fisher	1.09 (0.20-5.97)	1
Vitamin K antagonists	Chi-square	0.13 (0.05-0.33)	<0.001
DOACs	Chi-square	0.45 (0.19-1.11)	0.113
Direct thrombin inhibitors (DTIs)	Fisher	0.81 (0.21-3.09)	1
Platelet aggregation inhibitors	Chi-square	0.97 (0.62-1.53)	0.988
Aspirin	Chi-square	0.69 (0.46-1.05)	0.1
Hydroxychloroquine	Fisher	4.42 (1.10-17.79)	0.032
ACE inhibitors	Chi-square	0.66 (0.44-0.99)	0.057
ARBs	Chi-square	1.20 (0.80-1.78)	0.433
Acetic acid derivatives	Fisher	1.09 (0.32-3.64)	1
Oxicam derivatives	Fisher	2.19 (0.44-10.90)	0.386
Propionic acid derivatives	Chi-square	1.53 (0.58-4.07)	0.547
Coxibs	Fisher	2.19 (0.44-10.90)	0.386
Systemic glucocorticoids	Chi-square	0.42 (0.23-0.78)	0.007
Inhaled corticosteroids	Chi-square	0.66 (0.35-1.24)	0.251
Selective immunosuppressants	Fisher	0.87 (0.17-4.50)	1
Calcineurin inhibitors	Fisher	0.72 (0.14-3.60)	1
Other immunosuppressants	Fisher	1.46 (0.51-4.14)	0.576
Statins	Chi-square	0.83 (0.62-1.11)	0.228

Univariate associations with in-hospital mortality are detailed in Table 4. Several pre-admission medications were associated with an increased OR of death: heparins, VKAs, ARBs, and statins.

**Table 4:** Univariate analysis of prior medication and mortality * The estimate is based on six exposed patients. DOACs: direct oral anticoagulants; ACE: angiotensin-converting enzyme; ARBs: angiotensin-II receptor blockers; OR: odds ratio; CI: confidence interval

Predictor	Test	OR with CI (95%)	P-value
Heparin-like substances*	Fisher	11.88 (1.38-102.14)	0.011
Vitamin K antagonists	Chi-square	1.77 (1.10-2.83)	0.023
DOACs	Chi-square	1.67 (0.83-3.36)	0.207
Direct thrombin inhibitors (DTIs)	Fisher	1.34 (0.39-4.61)	0.742
Platelet aggregation inhibitors	Chi-square	1.30 (0.83-2.02)	0.3
Aspirin	Chi-square	1.92 (1.33-2.78)	<0.001
Hydroxychloroquine	Fisher	1.88 (0.50-7.06)	0.464
ACE inhibitors	Chi-square	1.25 (0.86-1.82)	0.286
ARBs	Chi-square	1.66 (1.12-2.44)	0.014
Acetic acid derivatives	Fisher	1.68 (0.53-5.34)	0.359
Oxicam derivatives	Fisher	0.47 (0.05-4.00)	0.675
Propionic acid derivatives	Chi-square	0.31 (0.07-1.35)	0.165
Coxibs	Fisher	0.47 (0.05-4.00)	0.675
Systemic glucocorticoids	Chi-square	1.59 (0.98-2.57)	0.079
Inhaled corticosteroids	Chi-square	1.05 (0.58-1.89)	0.997
Selective immunosuppressants	Fisher	0.93 (0.18-4.84)	1
Calcineurin inhibitors	Fisher	0.33 (0.04-2.71)	0.447
Other immunosuppressants	Fisher	1.57 (0.55-4.45)	0.402
Statins	Chi-square	1.71 (1.28-2.28)	<0.001

To ensure sufficient numbers for the multivariate analyses, medications within certain drug classes were combined into broader groups. Specifically, oral anticoagulants, including DOACs and DTIs, were consolidated into a single variable. Similarly, various types of NSAIDs were grouped into a single "NSAID" category. Systemic glucocorticoids and inhaled corticosteroids were also merged and classified as "corticosteroids." Finally, selective immunosuppressants, calcineurin inhibitors, and other immunosuppressants were combined into a single "immunosuppressants" group.

The results of the multivariate logistic regression analysis for ICU admission are presented in Table 5. After adjusting for confounders like patient characteristics and comorbidities, the pre-admission use of several medications was independently associated with lower odds of ICU admission: VKAs, DOACs, aspirin, and corticosteroids. Conversely, pre-admission use of HCQ and ACEIs was associated with higher odds of ICU admission. Regarding patient characteristics, both aged ≥65 years and female sex remained independently associated with lower odds of ICU admission. In the final model, no pre-admission comorbidities showed a significant association with ICU admission.

**Table 5:** Multivariate analysis of prior medication use and ICU admission Confounders included in the multivariable regression: medications (heparin-like agents, vitamin K antagonists, DOACs, aspirin, other antiplatelets, hydroxychloroquine, ACE inhibitors, ARBs, NSAIDs, steroids, immunosuppressants, statins), patient characteristics, and comorbidities (cardiovascular disease, diabetes, age ≥ 65, hypertension, sex, thromboembolism, pulmonary disease). DOACs: direct oral anticoagulants; ACE: angiotensin-converting enzyme; ARBs: angiotensin-II receptor blockers; NSAIDs: nonsteroidal anti-inflammatory drugs; OR: odds ratio; CI: confidence interval

Predictor	OR with CI	P-value
Intercept	0.89 (0.67-1.17)	0.3699
Vitamin K antagonists	0.11 (0.04-0.29)	<0.001
DOACs	0.43 (0.2-0.96)	0.031
Aspirin	0.57 (0.37-0.87)	0.01
Hydroxychloroquine	7.39 (1.7-32.14)	0.008
ACEI	1.60 (1.01-2.46)	0.031
Corticosteroid	0.51 (0.31-0.83)	0.007
Age ≥65	0.71 (0.52-0.97)	0.032
Female	0.54 (0.39-0.74)	<0.001

The multivariate logistic regression results for in-hospital mortality are presented in Table 6. In contrast to the univariate analysis, only pre-admission heparin use remained associated with higher odds of death in the multivariate model; however, its CI was wide, likely due to the small number of users. Regarding patient characteristics, being aged ≥65 years was associated with higher odds of death, while female sex was associated with lower odds. No significant associations were found between pre-admission comorbidities and in-hospital mortality in the final model.

**Table 6:** Multivariate analysis of prior medication use and in-hospital mortality * The estimate is based on six exposed patients. Confounders included in the multivariable regression: medications (heparin-like agents, vitamin K antagonists, DOACs, aspirin, other antiplatelets, hydroxychloroquine, ACE inhibitors, ARBs, NSAIDs, steroids, immunosuppressants, statins), patient characteristics, and comorbidities (cardiovascular disease, diabetes, age ≥ 65, hypertension, sex, thromboembolism, pulmonary disease). DOACs: direct oral anticoagulants; ACE: angiotensin-converting enzyme; ARBs: angiotensin-II receptor blockers; NSAIDs: nonsteroidal anti-inflammatory drugs; OR: odds ratio; CI: confidence interval

Predictor	OR with CI	P-value
Intercept	0.1 (0.07-0.16)	<0.001
Heparin-like agents*	21.76 (2.11-224.17)	0.009
Aspirin	1.39 (0.94-2.06)	0.094
ARB	1.42 (0.94-2.14)	0.097
Age >65	6.49 (4.22-9.99)	<0.001
Female	0.66 (0.47-0.92)	0.011

## Discussion

This study investigated the associations between pre-admission use of medications across multiple drug classes and two critical clinical outcomes in hospitalized COVID-19 patients: ICU admission and in-hospital mortality. Our findings, derived from multivariate models adjusted for patient characteristics and comorbidities, indicate that these associations are outcome specific. The set of prior medications linked to the likelihood of ICU admission was largely distinct from those associated with in-hospital mortality, underscoring the complex interplay between chronic therapies and COVID-19 progression.

Anticoagulants

Our cohort study examined three sub-classes of pre-admission anticoagulants: heparin-like agents, VKAs, and DOACs. After full adjustment in our multivariate models, we observed distinct associations. Pre-admission use of VKAs and DOACs was linked to significantly lower odds of ICU admission, with adjusted odds ratios (aORs) of 0.11 and 0.43, respectively. In contrast, pre-existing heparin-like therapy was independently associated with markedly higher in-hospital mortality (aOR = 21.8). Previous studies on the impact of anticoagulant treatment history on COVID-19 outcomes have had mixed results, while some studies suggest prior DOAC use has a protective effect on COVID-19 severity [17,24], others report no significant impact [25,26]. This discrepancy might stem from differences in study populations or, perhaps, the more specific drug classifications used in our analysis. For heparin, our observation that its pre-admission use was associated with increased odds of death aligns with some previous reports [27], although other studies have found no association [28]. The extremely high OR for heparin found in our study warrants cautious interpretation, as only six patients were using heparin and may reflect confounding by indication.

Antiplatelets

In our analysis of the antiplatelet group, only pre-admission aspirin use was independently associated with lower odds of ICU admission in the multivariate model (aOR = 0.57); other antiplatelet agents failed to show a significant effect. For the outcome of in-hospital mortality, aspirin was linked to increased odds in the univariate analysis. However, this association lost statistical significance in the fully adjusted multivariate model (aOR = 1.39, p=0.09). These findings reflect the inconsistent results observed in previous studies. While many reports find no association between prior aspirin use and worse COVID-19 outcomes [10-12,29,30], some have suggested an increased OR of death [27]. This contrasts with a meta-analysis indicating that aspirin might reduce the OR of death [31]. Consequently, the precise impact of pre-admission aspirin use on COVID-19 progression remains uncertain and likely depends on various patient and clinical factors.

Glucocorticoids

Our analysis combined both pre-admission systemic and inhaled corticosteroids into a single group. We found that the prior use of these medications was associated with significantly lower odds of ICU admission (aOR = 0.51), while no significant effect on in-hospital mortality was observed. Some previous studies have suggested that inhaled corticosteroids might be linked to decreased mortality [32,33]. Some studies report no significant effect of glucocorticoids [34], or even indicate that long-term use of systemic corticosteroids could increase the mortality and severity of COVID-19 [35-37].

RAAS inhibitors

Regarding RAAS inhibitors (including ACEIs and ARBs), our study found that pre-admission ACEI use was independently associated with higher odds of ICU admission (aOR = 1.6). In contrast, neither ACEIs nor ARBs showed a significant association with in-hospital mortality in the final adjusted model. These findings appear to differ from several previous studies, which generally reported no association between RAAS inhibitor use and severe COVID-19 outcomes, such as mortality [38-42], or even suggested a protective effect [43,44]. This discrepancy might be attributed to differences in study populations. While many earlier studies concentrated on patients with hypertension, our cohort encompassed patients with a broader spectrum of comorbidities, which could potentially alter the observed associations.

Hydroxychloroquine (HCQ)

In our multivariate analysis, pre-admission use of HCQ was strongly associated with higher odds of ICU admission (aOR = 7.4). While some studies, particularly those looking at patients with underlying conditions taking HCQ before infection, suggested potential benefits like reduced mortality [45], others found no benefit or even an association with increased adverse events and mortality, especially when used as a treatment for active COVID-19 [46,47]. Because only nine patients used HCQ in our cohort, the association with ICU admission should be interpreted cautiously and considered hypothesis-generating.

Immunosuppressants

We did not find any association between immunosuppressants (including calcineurin inhibitors, selective biologics, etc.) and ICU admission or in-hospital mortality. Previous studies on the effects of immunosuppressants have inconsistent results: some studies have found that immunosuppressants are associated with higher mortality [48] and ICU admission [49], while others suggest potential beneficial effects on mortality [50] and ICU admission [51]. The observed inconsistencies across studies likely arise from the heterogeneity of patients' underlying conditions for which these drugs are prescribed. This analysis is statistically underpowered (30 patients from a variety of immunosuppressants), resulting in wide CIs. The absence of an association should be interpreted as inconclusive rather than negative.

Statins

Our analysis found no significant association between pre-admission statin use and either ICU admission or in-hospital mortality. This finding is similar to the inconsistencies in the current literature; while some studies have reported no correlation between prior statin use and COVID-19 outcomes [9,52], others suggest an association with lower mortality rates [53-55].

NSAIDs

NSAIDs, a diverse class of medications, showed no significant link between their pre-admission use and either ICU admission or mortality. This lack of association aligns with several previous studies, which also concluded that prior NSAID use is generally not linked to worse clinical outcomes in COVID-19 patients [15,56].

ICU admission does not mirror mortality

In our study, in-hospital mortality was not significantly associated with ICU admission, suggesting that a higher ICU admission rate does not necessarily indicate a higher mortality rate. In our study, patients aged 65 years or over were less likely to be admitted to the ICU, while increased mortality was observed among this age group.

This may be due to the fact that during the first wave of COVID-19 in the Netherlands, ICU capacity was severely limited, and not all patients could be admitted. Dutch ICUs operated under formal triage frameworks that explicitly incorporated factors such as age and frailty when allocating scarce beds [57]. In addition, some older patients die (or are transitioned to palliative care) before an ICU transfer is contemplated. The distinction may help explain why antecedent medications were found to have differing associations with ICU admission and with mortality.

Strengths and limitations

The strength of our study is that, in contrast to many investigations focusing on single medications, it simultaneously evaluated numerous drugs across multiple classes. This approach enabled us to assess the independent associations of each medication with COVID-19 outcomes while adjusting for the use of other therapies. Furthermore, unlike studies that often restrict inclusion based on specific diseases, our cohort broadly included patients with various comorbidities. This inclusive methodology likely provides a more comprehensive understanding of these medication associations and enhances the generalizability of our findings to a real-world hospitalized COVID-19 population.

Our study has several limitations. The data we collected (February 20 to June 24, 2020) covered only the first wave of the epidemic - before the widespread use of steroids, antiviral drugs, or vaccinations. The EHR did not capture dose, duration, adherence, absence of pre-admission diagnoses, or prescribing indications for home medications may lead to bias. And our analytic cohort excluded patients without a documented pre-admission medication list and those with unknown age and medication history, which might lead to selection bias. The results may not be applicable to subsequent phases or the current epidemic response. The sample size also restricted our statistical power, particularly for analyzing medications with low prevalence. In addition, our retrospective data collection meant we lacked information like baseline oxygenation, and vaccination status was not available, residual confounding cannot be excluded. Finally, precise time-to-event data (e.g., time from admission to ICU or death) were not available, which made the survival analysis impossible.

## Conclusions

In conclusion, this study highlights the complex and often different associations between prehospital medication use and different severe outcomes in hospitalized COVID-19 patients. After adjusting for patient characteristics and comorbidities, medications whose associations with ICU admission differed from those with in-hospital mortality. This emphasizes the importance of considering specific outcomes and confounding factors when assessing the impact of prior medications. Although our study identified potential risk and protective factors, we cannot establish causality. More prospective studies that contain larger populations should be conducted, including on prescription indications, dosage, and adherence; and propensity score matching and causal inference should be applied to mitigate the effects of confounding factors.
